# People living with dementia and their family carers’ adherence to home-based Tai Chi practice

**DOI:** 10.1177/1471301220957758

**Published:** 2020-09-12

**Authors:** Yolanda Barrado-Martín, Michelle Heward, Remco Polman, Samuel R Nyman

**Affiliations:** Department of Psychology and Ageing & Dementia Research Centre (ADRC), 6657 Bournemouth University, Fern Barrow, Poole, UK Centre for Ageing & Population Studies, Research Department of Primary Care & Population Health, London, UK; Ageing & Dementia Research Centre (ADRC) and Department of Rehabilitation and Sport Science, 6657 Bournemouth University, Fern Barrow, Poole, UK; School Exercise & Nutrition Sciences, 72524 Queensland University of Technology, Australia; Department of Psychology and Ageing & Dementia Research Centre (ADRC), 6657 Bournemouth University, Fern Barrow, Poole, UK Department of Medical Science, Public Health, Bournemouth University, Fern Barrow, Poole, UK

**Keywords:** home-based practice, exercise, barriers, facilitators, dementia, carers, dyads, Tai Chi

## Abstract

**Objectives:**

The aim of this study was to understand what influenced people living with dementia and their family carers’ adherence to the home-based component of a Tai Chi exercise intervention.

**Method:**

Dyads, of people living with dementia and their family carers, who participated in the intervention arm of the Tai Chi for people living with dementia trial, were invited to join weekly Tai Chi classes for 20 weeks and practice at home. Semi-structured dyadic home interviews were conducted on average after 16 weeks of classes. The views of 15 dyads with a range of home practice adherence were sought in semi-structured interviews. The interviews were analysed using an inductive thematic approach.

**Results:**

Most participants found time to practise Tai Chi at home and practised for 18 hours on average. Amongst the barriers to adherence were participants’ competing commitments and a booklet not sufficiently conveying the Tai Chi movements. Hence, a video or DVD was requested by participants. Facilitators of their adherence to the home-based component of the intervention were their enjoyment of the practice and the development of a habit, which was supported by their commitment to the study and their willingness to benefit from Tai Chi.

**Conclusion:**

Enjoyment and perceived benefits had a great impact on participants living with dementia and their carers’ adherence to home-based Tai Chi practice. However, difficulties to perceive the Tai Chi movements through images might be hindering sustained participation. Hence, alternative aids such as videos and DVDs should be explored to facilitate adherence.

## Background

It is estimated that around 50 million people around the world are currently living with dementia (Alzheimer’s Disease International, 2015). Such prevalence makes dementia the second leading cause of disability amongst older adults, making it a public health priority (WHO, 2018). As dementia progresses the individual becomes more dependent on those supporting them such as family carers and friends, which impacts on their quality of life as well (Department of Health, 2015; WHO, 2018).

Falls are amongst the leading causes of death and dependency in older adults (WHO, 2007). People living with dementia are at a greater risk of falls (Shaw, 2003). Moreover, the consequences of falls in older adults living with dementia are worse than for their peers and include a higher dependence towards their family carers and risk of death (Allan et al., 2009; Fernando et al., 2017; Shaw, 2003).

Exercise interventions requiring balance, and Tai Chi in particular, are effective in preventing falls (Forbes et al., 2015; Gillespie et al., 2012). However, adherence to such interventions remains a challenge for older adults living with dementia (Burton et al., 2015). Yet the focus of Tai Chi studies so far has been to test their effectiveness rather than understand the barriers and facilitators for people living with dementia’s adherence (Barnes et al., 2015; Burgener et al., 2008; Yao et al., 2012).

In community-based exercise interventions aimed at people living with dementia, tailoring exercises to participants’ needs and environment (Pitkälä et al., 2013), as well as using written materials, memory aids (Logsdon et al., 2009; Prick et al., 2014) and exercise sheets to record practice (Suttanon et al., 2012) have been shown to facilitate home practice. Likewise, a lack of a practice companion such as a family member of friend can be a barrier for adherence in home settings (Suttanon et al., 2012). Studies using a dyadic approach, where both the person living with dementia and a family carer take part together, overcame this difficulty (Saravanakumar et al., 2014; Yao et al., 2012). However, despite the relevance of relationships in the context of dementia, joint dyadic interventions for people living with dementia and their family carers are relatively under used (Rausch et al., 2017) and, likewise, underexplored.

The development of interventions to sustain quality of life and reduce the impact of a progressively higher dependency on family carers, friends and the wider society are needed (Logsdon et al., 2007). Hence, understanding the aspects facilitating or hindering people living with dementia’s adherence to such interventions is of critical importance to optimise their participation and to reap the benefits. To achieve this understanding of how exercise behaviour can be promoted, there is a need to link research findings to theory that helps to explain exercise behaviour and what conditions lead to a higher exercise practice. However, this has rarely been done in previous exercise studies with people living with dementia (Yu & Swartwood, 2012). Nevertheless, existing theory such as self-determination theory (SDT), seems appropriate to understand people living with dementia and their carers motivational orientation to sustain exercise behaviour over time (Ingledew et al., 1998). SDT postulates that individuals are more likely to sustain behaviours they intrinsically find enjoyable, self-determined, and that contribute to the fulfilment of their basic needs of autonomy, competence and relatedness (Deci & Ryan, 2000). Similar to this competence, self-efficacy theory postulates that individuals’ behaviour results from the influence of their perceived ability to succeed in performing a behaviour (perceived self-efficacy) and what they perceive will achieve as a result of their behaviour (outcome expectancies) (Bandura, 1977). However, self-efficacy does not consider the different levels of autonomy in the individual’s motivation to continue practising. Hence, self-efficacy could be partially useful to explain initial adherence to the Tai Chi home practice, as it has been previously identified as a facilitator of exercise behaviour at the initial stages of practice (Lox et al., 2010), and SDT could help explain sustained Tai Chi practice at home.

This study aims to understand what influenced Tai Chi home practice adherence among people living with dementia and their family carers’ in the context of the TAi ChI for people living with demenTia (TACIT) trial (Trial Registration: NCT02864056) (Nyman et al., 2018). The TACIT trial is looking to explore the effects of Tai Chi in improving the postural balance of people living with dementia, which has been related to the prevention of falls. Hence, facilitating people living with dementia’s sustained participation in Tai Chi exercise practice at home could contribute to developing new healthy lifestyles as well as the enhancement of their well-being and quality of life. To our knowledge, this is the first study to explore people living with dementia and their carer’s experience of practising Tai Chi at home.

## Methods

### Participants

Participants were recruited between April 2017 and July 2018. Recruitment sources included three National Health Service Trusts, 15 General Practitioner surgeries, the Join Dementia Research Website, the Alzheimer’s Society and publicity (via flyers or face-to-face events attended by Bournemouth University Team) across three different research sites in the South of England. A total of 359 participants were referred and screened by the Bournemouth University Team, amongst those 86 were randomised after being found eligible and willing to take part in the trial. Of these 86 dyads, 42 dyads were allocated to the intervention arm and were divided into 10 different groups.

This study sample represents participants allocated to the intervention arm of the first six out of the 10 groups organised (*n* = 22 dyads) who participated in the trial and received the instructor’s home visit. Participants in groups 7–10 could not be included in this qualitative study due to time and funding restrictions, once the trial received an extension to recruit more participants. Groups differed on the geographical area where they were based, number of participants allocated (with a minimum of three dyads and a maximum of eight dyads), dates and times of the sessions and the instructor (instructor 1 or instructor 2) delivering the class-based component of the intervention. Demographic characteristics of participants included in this study are provided in Table 1 and inclusion and exclusion criteria for participants in the TACIT trial are described in Supplementary Material A as reflected on the paper reporting on dyad’s experiences of taking part in Tai Chi Classes (under review elsewhere). A total of 25 dyads were initially allocated to groups 1 to 6; however, not all managed to attend the first class due to withdrawing after randomisation and prior to the start of the classes (*n* = 1) or due to health issues that impeded the person living with dementia to attend any of the classes (*n* = 1), or to do any home practice as they withdrew before receiving the instructor’s visit (*n* = 1). As such, only 22 dyads were observed during the classes, and of these, 15 dyads were interviewed.

**Table 1.** Participants’ characteristics.

Participant	Item	Frequency or mean (SD)
People living with dementia	Gender	
	Male	12
	Female	10
	Age, mean (SD)	79 (6.46)
	Relationship status	
	Married/civil partnership	19
	Single	1
	Divorced	1
	Widowed	1
	Current living situation	
	Living with family/friends	21
	Alone	1
	Level of education	
	None	1
	Primary	1
	Secondary	13
	Higher education college/university	5
	Further education/professional qualification	2
	Ethnicity	
	White	22
	Dementia type^a^	
	Alzheimer’s	15
	Mixed Alzheimer’s and vascular	6
	Other (frontal lobe)	1
	Months diagnosed with dementia, mean (SD)	18.4 (18.1)
	Other chronic conditions	
	Yes	17
	No	5
	Existing injuries or health injuries to beconsidered to do Tai Chi	
	Yes	5
	No	17
	Use of walking aid	
	Yes	6
	No	16
	Prescribed daily medications, mean (SD)	5.45 (3.62)
	Falls in the last year	
	Yes	9
	No	13
	Falls in the last month	
	Yes	4
	No	18
	Frequency of moderate PA practice	
	Everyday	11
	3 times per week	3
	2 times per week	1
	Monthly	1
	Rarely/never	6
	Frequency of vigorous PA practice	
	Everyday	1
	Weekly	2
	Rarely/never	19
	Previous experience practising Tai Chi	
	Yes	4
	No	18
	M-ACE score, mean (SD)	16.4 (4.92)^b^
Carers	Gender	
	Male	6
	Female	16
	Age, mean (SD)	72.4 (9.48)
	Relationship with the person livingwith dementia	
	Spouse/partner	18
	Son/daughter	2
	Brother/sister	2
	Live with the person living with dementia	
	Yes	19
	No	3
	Relationship status	
	Married/Civil partnership	20
	Single	1
	With partner	1
	Current living situation	
	Living with family/friends	22
	Living alone	0
	Level of education	
	Primary	2
	Secondary	11
	Higher education college/university	5
	Further education/professionalqualification	3
	Missing data	1
	Ethnicity	
	White	25
	Previous experience practising Tai Chi	
	Yes	2
	No	20

PA: physical activity; SD: standard deviation.

^a^Dementia diagnosis was provided by healthcare providers supporting the study (NHS sites) with participants’ consent.

^b^Mini Addenbrooke’s Cognitive Examination (M-ACE) (Hsieh et al., 2015) scores range from 0 to 30, where higher scores reflect better cognitive performance. Participants were not included in the trial if their score was nine or lower, as this was indicative of severe dementia.

### Design and instruments

As part of the trial, at baseline, participants’ demographic details were collected through a structured questionnaire (see Table 1). A qualitative approach was later used to explore dyads’ experiences of taking part in the TACIT trial. A semi-structured schedule was used to guide interviews and gather dyads’ experiences (see Supplementary Material B).

### Procedure

Participants in the intervention arm were invited to join weekly Tai Chi classes for 20 weeks, as well as practising at home for 20 minutes a day, as per protocol (Nyman et al., 2018). Classes were held once per week in venues local to the dyads and lasted for 45 minutes, followed by time for socialising. During these classes, dyads were encouraged to practice Tai Chi at home following a home visit from the instructor within the first 3 weeks of classes. Dyads were asked to practice at home for 20 minutes a day on days with no classes. The classes were led by two fully trained and experienced Tai Chi instructors, who were in charge of making a home visit with each dyad after their second week attending classes.

Around week 16 of their involvement in the study, 15 dyads out of the 22 participating in the six first groups organised were invited to take part in a dyadic interview at home. A purposive sampling strategy ensured the inclusion of a range of participants with different characteristics, for example home practice adherence over weeks 3–15, age, gender and dyadic relationship. All dyads invited to take part in an interview agreed to take part together at their home. Interviews were audio recorded and professionally transcribed verbatim.

### Tai Chi intervention at home

During instructors’ home visits, several behaviour change techniques were used to promote exercise practice as per protocol (Nyman et al., 2018). Dyads completed an action and coping plan with the instructor, who left a copy of these at participants’ home for their reference. The action plan reflected what days of the week, when, where and for how long would participants be practising at home; whilst the coping plan was designed to anticipate barriers to their home practice as well as ways of overcoming such challenges. Similarly, during these initial home visits, instructors provided participants with an alarm clock to remind their practice at their agreed time of practice and 18 exercise logs to report their weekly home practice.

At the end of the group sessions, participants were provided with a total of three colourful, professionally produced, illustrated booklets as well as nine additional sheets designed to point participants towards the exercises to be practised each week. Participants were due to practise at home according to the week number, with some additional sheets covering several weeks.

### Ethical considerations

The trial and this study was ethically approved by the West of Scotland Research Ethics Committee 4 (reference: 16/WS/0139) and the Health Research Authority (IRAS project ID: 209193). Participants were provided with a participant information sheet by post halfway through their involvement in home practice and given a minimum of 48 hours to consider their participation in the interview. It was made clear to the participants that their participation was voluntary and independent from their involvement in the TACIT trial. Hence, an additional written, informed consent was obtained from both members of the dyad participating in the TACIT trial and wanting to participate in this study. Additionally, a process consent was followed, paying attention to participants living with dementia’s engagement throughout the interview to ensure their willingness to continue (Dewing, 2008).

### Data analysis

Interview transcriptions were coded in Nvivo 11 (QSR International Pty Ltd., 2012) thematically. Each interview was coded promptly to afford refinement of the interview probes for subsequent interviews until data saturation was reached. The six steps described by Braun and Clarke (2013) were used at this stage to get familiarised with the data, generate initial codes, search for themes, review those initial themes, define the themes and write up the report. Data were inductively coded by the first author and 10% of the data were double coded by the second author following a coding manual developed by the first author, reaching a strong level of agreement in the quotes being coded under the same codes (Kappa value: 0.90). Finally, resulting themes and codes were reviewed by all four authors. Authors discussed themes and codes through regular meetings while data were being analysed and adjustments were made by the first author; however, no major disagreements were found during this iterative process.

## Results

Generally, participants found time to practise Tai Chi at home; however, this time did not always reach the recommended dosage of 50 hours over the 5 months intervention period. Adherence to the classes (reported elsewhere) was better than home practice, and home practice was higher among those that attended more classes. Overall, participants living with dementia practised at home an average of 18 hours (1097 minutes) throughout their participation in the study ranging from 0 to 61 hours (0–3650 minutes) as reported in Supplementary Material C. That is people living with dementia received an average of 54% (range 0–179%) of the recommended home practice dosage since their recruitment. Carers practised at home an average of 20 hours (1220 minutes) ranging from 0 to 61 hours (0–3650 minutes), achieving 60% (range 0–179%) of the recommended home-practice dosage since they received the instructor’s visit. However, data accuracy was reduced as some participants returned only a few exercise logs, particularly when they missed a class. With the data available, on average each dyad undertook approximately 86 minutes (95% CI , 63–109) practice per week from a target of 120 minutes per week.

A total of five themes were identified around dyads’ experiences of practising Tai Chi at home. These are reported below in order of themes with highest number of quotes first: Supportive materials, behaviour change elements, ways of practising, barriers and facilitators.

### Supportive materials

Most dyads did not report on their use of the sheets provided to guide their practice; however, when five dyads referred to them during the interviews, they found them useful. Having said that, two carers pointed that the contents described would not fit the 20 minutes recommended practice.

*I mean, there’s no way, there’s absolutely no way you could do all that in twenty minutes [laughs]. … I’ve altered it, but it says warm up eight times, we only do it four times because warm up eight times would probably take about fifteen minutes [laughs]* (01002C^1^).

Six participants found that the booklet worked well for them with no additional information required. Others reported that the pictures and description of the movement patterns were difficult to comprehend: ‘…*you can’t really show how it got there and how it’s going to go on. So, it’s really difficult to portray movement in a, in a stationary picture* (01055P)’, *‘Well, easy, when you were looking at it, but not so easy when you were trying to do it at the same time as looking at it [laughing]*’ (01009C).

Additional visual support was particularly missed at the early stages of home practice. Once participants were familiar with the movements, some felt the booklet was not needed anymore. Others reported it was good for reminding them about the movements but felt that attending more sessions and getting more familiar with the movements would be more beneficial instead. In most dyads, carers read the booklet and guided the person with dementia’s practice at home, this could be due to some people living with dementia’s reading difficulties:

*Because I’ve got dementia, I don’t follow the book. <01008C> tells me what to do. Because it would be too…take up too much time for me to follow the book. And I get, um, [pause] muddled…sometimes about reading* (01008P).

When participants were asked about ways their home practice could be facilitated, they either reported no suggestions or proposed improvements to the booklet. The most popular request was the need to produce a video or DVD complementing the booklets. This way they could have ‘someone showing them all the time’. When prompted, only a few mentioned that a DVD would not be required because the booklet was sufficient or they had no DVD player. Five dyads felt that a DVD would be a better alternative to guide their practice and overcome their initial insecurities when practising at home, whereas others pointed that this would make home practice more attractive to those who are not so eager (03008) and that it could make it easier for them to practise longer periods (45 minutes) as they did in the classes.

*I think a DVD or something, if you’re doing it along to something, I think that would be good. You know, like <Instructor 1> there (…) rather than standing looking at a book and looking at your wall. It’s harder, it’s…it’s not as enjoyable [as the classes], obviously. (…)…unless you’re sort of like completely addicted to Tai Chi, which obviously some people are…and that’s what they do all the time [laugh], people I know, um, then they will just meditate and do their Tai Chi, whereas we’re looking at the wall* (03008C)*.*

Failure to meet such need to further support their home practice might translate into dyads seeking alternative support without consultation with the instructors. For instance, two dyads admitted having looked at YouTube videos to support their practice, although they realised the movements shown were not the same as the ones they had been taught (01039, 01021). For this reason, one of the carers decided to wait and postpone the use of these videos until their participation in the study had finished. Another carer reported having bought a beginners Tai Chi DVD to continue their practice after the study period.

The suggestions to improve the booklet were as follows: More pictures or diagrams showing the progression of the movements, more accurate and detailed explanations for beginners and improving the grammar. An additional suggestion was to transform the booklets in A3-size flips that could stand on their own, so participants could look at them easily when performing the movements.

### Behaviour change techniques to support home practice

#### Instructors’ home visits

Although two carers did not value the home visit of the instructor, others indicated that the visit showed genuine interest and ensured safe practice. Despite the mixed reaction to the home visit, none of the participants suggested any improvements to these home visits to make them more useful to them and their practice: *‘Um, well she…it was just the explanation and what it was all about and what we were going to be doing. And it sounded really interesting and…we’re game for anything really. And it’s all in a good cause…’* (01021C)*.*

#### Action and coping plans

When asked during the interviews 14 out of 15 dyads reported that they had not used the action and coping plans: ‘*Something I’ve filed and... [laughs]. I didn't file it under “b” for bin I filed it in “bag” [laughs]*’ (01002C). Only one pointed out that the coping plan was useful as they could think about what to do when they did not feel well. Some participants reported that they had not faced any difficulty to find the time to practise, or that they did not practise when they planned to do it, or need to adapt their practice to their other commitments: ‘*I don't [use them], because I just have to, erm, play it by ear, really...as to when we can fit in the, erm, the…[practice]*’ (01009C). During the interviews, some realised that they had not done as much practice as they initially planned but managed to do as much as they could instead, or reported having experienced unexpected difficulties (including adverse events and serious adverse events unrelated to their Tai Chi practice) that had an impact on their adherence: ‘*We haven’t found any difficulties [finding time to practise at home, as per coping plan]. You know, everything changed with the <serious adverse event 1>, up to then and that’s the period we should really look at…*’ (03005C). Other participants who did not check the initial plan managed to practise longer and exceeded the recommended dosage (03006) or practised at different times than reported.

#### Clock

Only a few dyads commented on the use of the clock provided and none of them used it as an alarm clock to remind them when they were due to do their practice (i.e. to set a time for their practice). Merely having the clock around their home reminded one of the participants with dementia to practice, whilst three other dyads used it to check the length of their practice instead: ‘*No, no [we did not use the clock for reminding us of our practice], [we] just…just [used the clock] to time us for the…doing the [Tai Chi practice], you know, I find half an hour is…is right*’ (03006C-I). However, a couple of dyads reported their alarm clock was not functioning and another carer said it would be disturbing to have an alarm reminding her of doing their home practice.

#### Home-exercise logs

Among dyads participating in the first six groups organised (*n* = 22), the return rate of home exercise logs was 70% (266 received/368 expected). Of the interviewed dyads (15/22), most of them commented on the home-exercise logs. It was mainly the carers who completed these logs. Participants who experienced a serious adverse event and missed a class, frequently forgot to hand in their exercise logs. Eight dyads who regularly completed logs reported that it was beneficial in raising awareness of their practice and increased motivation to attend upcoming sessions, whereas four other dyads reported having completed them to just to comply with research procedures and felt it was more a ‘a bit of a bind’ (02002C).

*No, it is quite useful, it is...because you look at it and you think, oh, didn’t do any maybe we ought to do some [laughs]. Or we only did fifteen minutes, so we’ll do a bit more today. (…) Well it’s quite nice, it’s like doing the job and ticking it off isn’t it, you know how satisfying that is. So, at the end of it I write down twenty minutes and then this little halo comes here, and I go off and do other stuff [laughs]* (01002C)*.*

Two participants wondered during the interview if other participants would be reporting their honest time of practice in the forms and whether the instructor would be able to pick up on this, and they stated that they had been honest. During another interview, the person living with dementia unexpectedly shared that she had lied in one of the forms and she was not ashamed of it: ‘*I’m not ashamed that I did, because I thought, cause I look like I’m just ignoring it, so I just put twenty minutes, five minutes. (…) Not in that hot weather, I didn’t [practice at home], no*’ (01036P)*.*

### Ways of practising

Among those who returned their exercise logs as requested, those who adhered better to the home-based practice expressed they enjoyed it and had no major difficulties in finding time to practise at home. This was an opportunity to work together on practising the movements to become familiar with them and work on the weak points identified by the instructor during the classes.

#### Preferences

Dyads had different preferences in terms of how to implement their home practice. Some preferred mornings around breakfast (01039) whilst others ended up practising quite late in the evening (01022). Likewise, according to their needs, some practised in 10-minute blocks (01036C) whereas others practised 20 minutes or more continuously (01036P). Five dyads reported practising between 20–30 min/day or 30–40 min/day, trying to fit Tai Chi in where possible. For some, this was done at a regular time slot of similar duration, whereas for others the timing and duration varied and even went over the recommended daily dosage to improve a certain movement to fit in the content in the additional sheets provided or because with practice they felt they could handle longer periods: ‘*I actually did forty minutes yesterday, and I didn’t, it just went…because I was trying to do that new one, you know. And I kept thinking, I’ll do that again, I’ll do that one more time*’ (01036C).

Most participants developed a routine for their home-based practice. However, this was interrupted occasionally by having visitors, travel or competing commitments such as caring for other members of the family. Participants adapted home practice to their diverse needs. Some developed strategies to make home practice easier by involving a formal carer, family members (i.e. grandchildren) or friends when they were together during the Tai Chi practice time. Others used a mirror to facilitate home practice or used music to keep their practice more relaxed and focused and executing the movements slower.

*…And granddaughter would join in sometimes. But she’s four so you can imagine. But, um, if we said to the family, you know, we’re going to practise our Tai Chi then they left us to practise the Tai Chi and then <Granddaughter of 03005> decided that she was going to join in* (03005P).

#### The role of routine

Participants acknowledged the importance of developing a routine to fit Tai Chi into their daily life. Participants realised that a routine was beneficial to avoid procrastination and ensure daily practice. Whereas one group of participants incorporated Tai Chi as a daily routine to enhance its mastery and enjoy it for its intrinsic value, another group was driven by external motives. The latter, although they incorporated Tai Chi in their daily life, it was to get it over and done with: ‘*It’s almost another chore to be done, isn’t it?* (01022P).

*So I think really it is quite important that you do that and to me it’s quite important that you say every day, because then if you miss a day at least you…that’s really naughty, but you just think, oh well, you as the…think, well, we have got x amount of time out of them…(…) …so that’s good, yeah* (01025C)*.*

Whereas for a few participants home practice was motivated by external factors, others carried on even when on holiday or not at their home. Even one dyad opted to practise in public on different occasions: 01039P: ‘*And we have been out on the heath a couple of times as well so, which is*…’; 01039C: ‘*Yes, doing it in the fresh air, which is lovely isn’t it?*’

#### Progress over practice

Progressively, with practice, participants felt they had learnt ‘a lot’ or that the person living with dementia was able to remember the movements at home, occasionally anticipating the carers’ movements. Difficulties in remembering movements whilst practising at home might have impacted one dyad’s practice, which ended up doing the movements faster than they had been taught to avoid forgetting the sequences of movements after reading them in the booklet. This did not allow them to keep their ‘heads up’ and get the benefits of relaxation. However, the carer was confident that once they had learnt the sequences by heart, they would be able to adapt their speed and get additional benefits from Tai Chi:

*…so, he can see he’s beginning to remember it. So, I think doing it at home really helps that way. But I just need to get…make sure I’m doing the right sequences that follow it. Once I’ve got them mastered, we’ll be fine* (01021C)*.*

### Barriers to home practice

Despite most participants being able to find time to practise at home, some reported different reasons that might have reduced their practice: (a) not enjoying the home-based exercise, describing it as a ‘chore’ (01022 and 03008); (b) competing commitments (01012C and 02002C); (c) experiencing the consequences of previous or unexpected and unrelated health issues that impacted on both their class attendance and home practice (03005, 01008, 02002P, 03003 and 01055) or occasionally not feeling well (03006 and 01022); (d) occasional forgetfulness or procrastination, when saying ‘We will do it later’ and then ‘the later never comes’ (01025, 01055 and 01039); (e) having a busy day with family and friends over; (f) travelling or holiday and (g) dyad’s members not living together (01009, 01012 and 01036).

### Facilitators to home practice

Participants also pointed towards different factors as facilitators or strengths of the home practice: (a) the enjoyment of their home practice and having a laugh whilst practising at home (03005, 01039, 01025, 01055 and 01021); (b) the expectations of getting better at or achieving benefits of Tai Chi through repeated practice (01036C, 01055, 01008, 01009, 03006, 01021C and 02002C); (c) the development of a habit and Tai Chi being included in their daily routines (01009, 01022, 03006, 01002, 01021, 01039 and 01025); (d) doing it together, where the carer provides support and reminds about practice time (03006P, 01002, 01021, 01025, 01055 and 01039) and with the possibility of involving other family members or formal carers (03005 and 01036C); (e) having reminders around the house such as the exercise booklets (03006P) and clock (03006C and 01009P); (f) feeling that if any difficulty was identified it could be clarified with instructor in the following class (02002C); (g) feeling committed to the study (01021P, 01022 and 01039) which in one of the dyads translated into the person living with dementia encouraging their carer to practise at home and (h) the possibility of carrying the booklets when they go on holiday or do their practice somewhere else (01039 and 01002C).

## Discussion

The aim of this study was to understand what influenced adherence to the home-based component of a Tai Chi exercise intervention among dyads formed of people living with dementia and their family carers. To our knowledge this is the first study to explore their experiences of taking part in a home-based Tai Chi intervention as well as the elements supporting or hindering their practice.

Most participants found some time to practise Tai Chi at home, although their adherence to the recommended dosage was varied, with some dyads hardly doing any practice and others going well over the recommended dosage. Participants started practising at home after the instructor’s home visit to improve their performance and get the benefits of their practice. According to SDT (Deci & Ryan, 2000), these external forms of regulation are common when adopting a new behaviour like Tai Chi. In fact, at those initial stages, self-efficacy theory (Bandura, 1977) can help with understanding the initial persistence in Tai Chi practice when participants did not feel competent in performing the Tai Chi movements. At that stage, participants felt they would be able to remember the movements through repeated practice (mastery experiences), which was stressed by the instructors during the classes (verbal persuasion). During classes, instructors emphasised that movements could feel and look strange to the participants and that they also struggled when they started their practice years ago. Hence, having an instructor who served them as a model as their classmates (vicarious experiences) and who reminded them of the benefits associated to Tai Chi practise (verbal persuasion) could have contributed to their initial adherence to the home practice until they stated perceiving their improvement (mastery experiences) and experiencing some benefits. The initial perseverance was likely a contributor to perceiving Tai Chi as an enjoyable activity and becoming intrinsic motivated resulting in sustaining their home-practice regime (Ingledew et al., 1998).

Supportive materials were not sufficient for some dyads who missed more realistic sheets to guide their 20 minutes practice or additional visual cues (complementing the booklet) to practise independently at home at the early stages. Behaviour change techniques were not used as intended or valued by all participants as changes in time availability played a major role in their practice. Only home-exercise logs served participants as a motivator. Dyad’s practising regime depended very much on their preferences and time availability. However, adherence was better when dyads realised the importance of a routine and felt the progress over time due to their practice efforts. This likely resulted in enhanced feeling of competence which in turn might have facilitated the internalisation process outlined by SDT (Deci & Ryan, 2000) which resulted in participants finding solutions to barriers including having visitors or going away on holiday (Sebire et al., 2018).

### Adherence barriers

Main barriers for participants’ adherence to their home practice were additional commitments which clashed or reduced time availability and health issues affecting dyads. Previous literature also found competing commitments such as caring for other members of the family (Connell & Janevic, 2009; Farran et al., 2008) or a lack of time (Meyer et al, 2016) among factors that hindered participants adherence. One of the main weaknesses of home practice, however, was the lack of additional visual instruction material (DVD) which could have facilitated the development of self-efficacy beliefs by following a model as reported in previous studies (Hoogerheide et al., 2018; Van Gog & Rummel, 2010). Furthermore, despite using additional behaviour change techniques to facilitate adherence as advised by studies included in a recent review (Nyman et al., 2017), participants did not use such techniques as intended.

### Adherence facilitators

There were three main factors contributing to participants’ adherence to their Tai Chi practice at home: their enjoyment of their dyadic practice, feelings that they could end up mastering Tai Chi through repetition (by developing a habit) and experienced or expected well-being improvement attributed to their practice. Previous research has reported that enjoyment of the home-based component (Gonçalves et al., 2017; Wesson et al., 2013), together with participants self-efficacy beliefs, and feeling the benefit of the intervention (Escolar-Reina et al., 2010) facilitated adherence. In this study, home practice also allowed people living with dementia to have more individualised support from carers (acting as role models) to develop motor memory whilst practising the movements they found more difficult during the classes as previously reported in other exercise interventions (Barnes et al., 2015; Day et al., 2016; Suttanon et al., 2012). However, even when participants did not feel very competent at the early stages, those who were able to practise in dyads shared a laugh, in a more relaxed environment, which highlights the critical role of enjoyment. Additionally, habit theory might be able to explain the routine developed by dyads with better adherence (Jansons et al., 2018; Meyer et al., 2016; Verplanken & Aarts, 1999). Although adherent participants in the present study reported to have developed such habit, previous research has also indicated that reliance on self-report might result in overestimation of its effect (Gardner et al., 2011), which would require these findings to be taken with caution. As found in previous research, as the weeks passed, participants became aware of their progress and the easiness in remembering the movements enhancing, in turn, their self-efficacy beliefs (Campbell et al., 2001; Essery et al., 2017; Pozehl et al., 2010). This research also suggests that the use of more than one theory in an intervention may be more effective in this population.

### Strengths and weaknesses

The key strengths of this study are the inclusion of participants with different adherence levels to the home-based component, including participants who withdrew from the classes, and the use of dyadic interviews over other methods to explore their experiences. These strengths allowed the inclusion of diverse views and experiences of home practice and a joint reflection in a familiar and relaxed environment that could have facilitated more honest responses regarding their feelings towards the intervention. Among the limitations of this study, adherence to the home-based component was not objectively measured, and so there was a risk of participants over-reporting (Gardner et al., 2011) to avoid feeling bad when handing the exercise logs to the instructor. Likewise, by not monitoring what people actually did at home, it is difficult to establish how participants practised the exercises and whether they followed each weeks’ instructions. Finally, 11 out of the 22 dyads allocated to the intervention self-reported engaging in moderate physical activity (e.g. walking) on a daily basis before joining the study. This might have influenced their adherence to Tai Chi practice at home and their perception of barriers and facilitators, which could impact on this study finding’s transferability to older adults with dementia who may not be as physically active.

### Implications for research and practice

This is the first study to explore the experiences of those living with mild-to-moderate dementia and their carers practising Tai Chi at home. This exploration is relevant in the context of exercise research for people living with dementia as home practice might support the development of a habit as well as the maintenance of practice beyond the study period and reduce the costs of delivering an intervention in person.

This study implications for practice highlight dyads’, in the absence of an instructor, need of further support at home, preferably in the form of a visual medium (DVD, video and online) in the early stages. Whereas most of the dyads enjoyed the class-based component, home-based practice was only enjoyed by some. Considering the importance of developing exercise routines, any strategy that might be perceived as attractive (e.g. visual materials) should be tested to promote adherence at home, even if these do not translate into the development of a tight routine but one that allows the flexibility needed by participants (e.g. allowing an adaptation to the daily fluctuating needs of the person living with dementia or other caring responsibilities of family carers).

This study findings are part of a hybrid intervention (using both class and home-practice), which makes the lessons learnt at home applicable to interventions designed to be delivered only at home. From this study’s findings, it would be expected that in the context of a home-based intervention only, people living with dementia and their carers would need further guidance through a model (e.g. instructor) at least in the initial sessions, perhaps by using new technologies such as skype or video calling. Since participants appreciated instructor’s corrections and feedback, live sessions might then be more appropriate; however, the acceptability and accessibility of such technological methods should be explored as some participants in our study reported they would have not been able to use them.

## Conclusion

This study provides an overview of participants’ experiences regarding home practice and points the main barriers and facilitators to participants’ adherence to Tai Chi over a period of approximately 5 months. Amongst the most common barriers to adherence were participants’ other commitments and the booklet that was not easy for them to follow due to a lack of illustrations/DVD. Amongst the most common facilitators were the enjoyment of the practice and the development of a habit, which was supported by their commitment to the study and their willingness to benefit from Tai Chi.

## Supplemental Material

Supplementary_Material – Supplemental Material for People living with dementia and their family carers’ adherence to home-based Tai Chi practice Click here for additional data file. Supplemental Material, Supplementary_Material for People living with dementia and their family carers’ adherence to home-based Tai Chi practice by Yolanda Barrado-Martín, Michelle Heward, Remco Polman and Samuel R Nyman in Dementia

## Note

1. Participant identifiers include a number which identify the dyad, when a “P” is added at the end it refers to the person living with dementia only and a “C”, to the family carer only.
