# Supplementary material for: People living with dementia and their family carers’ adherence to home-based Tai Chi practice
Source: Dementia (London). 2020 Sep 12;20(5):1586–603. doi: 10.1177/1471301220957758 (PMC8216316; doi:10.1177/1471301220957758)
Supplement: Supplementary_Material – Supplemental Material for People living with dementia and their family carers’ adherence to home-based Tai Chi practice [file Supplementary_Material.docx]

**Supplementary Material A**

Table A1. Inclusion and exclusion criteria for the TACIT Trial

|  | **Inclusion criteria** | **Exclusion criteria** |
| --- | --- | --- |
| **For people living with dementia** | - A diagnosis of (mild to moderate) dementia; - Aged 18 years or older; - Living at home in the community (rather than residential care); - Able to practice standing Tai Chi; and - Have a carer available who would provide support during the assessments and during the group-based Tai Chi classes. | - A diagnosis of Lewy Body dementia or Parkinson’s disease; - Receiving end-of-life care; - Severe dementia according to the MiniAddenbroke’s Cognitive Examination (Hsieh *et al.*, 2015) (cut-off point M-ACE < 10); - Severe sensory impairments; - Already practicing Tai Chi, similar exercises (i.e., Qigong, yoga, or Pilates) or attending balance exercise programs (e.g., Otago classes); - Under the care or who had been referred to a falls clinic for assessment; - Unable to attend weekly classes; or - Lacking mental capacity to provide informed consent. |
| **For carers** | - Living with the person living with dementia or able to visit him/her at least twice per week; - Availability to support the person living with dementia by participating in data collection throughout the trial and in the intervention (if allocated to the intervention group); - Ability to do standing Tai Chi; and - Willingness to attend weekly Tai Chi classes. | - Presence of severe sensory impairment; or - Lack of capacity to provide informed consent. |

**Supplementary Material B**

Table B1. Interviews Schedule: Home-Based Practice Experiences

| **Question** | **Probes** |
| --- | --- |
| Tell me about your experience of doing the Tai Chi exercise at home.  [Prompt: Booklet] | Enjoyment:  ▪ What did you like about the Tai Chi exercises at home?  ▪ What did you not like about the Tai Chi exercises at home?  Participation:  ▪ What helped you to take part in the Tai Chi exercises at home?  ▪ Did anything stop you from taking part in the Tai Chi exercises at home?  Home visit by Tai Chi instructor:  ▪ How did you feel about the home-visit by the Tai Chi instructor?  ▪ What made it particularly useful?  ▪ What could be done to make it more useful?  Booklet:  ▪ How did you find the booklet as a guide to doing the Tai Chi exercises at home?  • Was it easy to follow?  • How could we improve it?  Improvements:  ▪ How could the home exercises be improved to make it easier for you to participate? |
| Many people find it difficult to carry on doing Tai Chi every week for several weeks. How would you describe the time you have invested in the Tai Chi exercise? | [Prompt: log of Tai Chi practice at home]  ▪ Have you been able to practise Tai Chi at home as frequently as you planned?  ▪ What, if anything, made it difficult to practise Tai Chi at home sometimes?  ▪ What helped you keep doing Tai Chi at home every week?  ▪ What was it like completing the weekly exercise log (Tai Chi exercise at home)?  i. What was it about the weekly logs that made them useful?  ii. How could we improve the weekly logs?  [Prompt: Action and coping plans]  ▪ How do you feel about the action plan that you did for practising Tai Chi at home?  ▪ What things helped you keep to your plan?  ▪ What things made keeping to your plan difficult?  ▪ Person with dementia – Tell me about how you used your coping plan  ▪ Carer – Tell me about how you used your coping plan  ▪ Has your perception about Tai Chi changed since you started practising it? In what way? |

**Supplementary Material C**

Table C1*. Participants’ Adherence to Home Practice (Minutes and Averages per Participant)*

|  |  |  |  |  |  |  | |  |  |  |  |  |  |  |  |  |  |  |  |  |  |  |  |  |  |  |  |  |  |  |  |  |  |
| --- | --- | --- | --- | --- | --- | --- | --- | --- | --- | --- | --- | --- | --- | --- | --- | --- | --- | --- | --- | --- | --- | --- | --- | --- | --- | --- | --- | --- | --- | --- | --- | --- | --- |
|  |  |  |  |  |  | **Person living with dementia (PLWD)** | | | | | | | | | |  | |  | | **Carer** | | | | | | | | | | | | |  |
| **Dyad number ^1^** | **Withdrew?** | **Week when they were offered to join the classes** | **Instructor's home visit was conducted after session number…** | **If withdrawn - number of their last session** | **Interviewed?** | **Total minutes of class practice (PLWD)** | | **Total minutes of home practice** | | **Average home practice since instructor's home-visit (weekly dosage=120min)** | | **PLWD's average home-practice (out of 35hrs recommended)** | | **PLWD's overall hours of Tai Chi until Final Follow Up home-visit)** | | **Average dosage received by the person living with dementia (out of 50hrs)** | |  | | **Minutes of class practice (Carers)** | | **Minutes of home practice** | | **Average home practice since instructor’s home-visit (weekly dosage = 120min)** | | **Carers' average home-practice (out of 35hrs recommended)** | | **Carers' overall hours of Tai Chi (until Final Follow Up home-visit)** | | **Average dosage received by carer (out of 50hrs recommended)** | |  |  |
| 03003 | No | 1 | 3 |  | Yes | 270 | | 220 | | 11% | | 10% | | 8 | | 16% | |  | | 270 | | 220 | | 11% | | 10% | | 8 | | 16% | |  |  |
| 03005 | No | 1 | 2 |  | Yes | 405 | | 445 | | 21% | | 21% | | 14 | | 28% | |  | | 405 | | 445 | | 21% | | 21% | | 14 | | 28% | |  |  |
| 03006 | No | 1 | 3 |  | Yes | 855 | | 3650 | | 179% | | 174% | | 75 | | 150% | |  | | 855 | | 3650 | | 179% | | 174% | | 75 | | 150% | |  |  |
| 01002 | No | 1 | 3 |  | Yes | 765 | | 2360 | | 116% | | 112% | | 52 | | 104% | |  | | 765 | | 2250 | | 110% | | 107% | | 50 | | 101% | |  |  |
| 01008 | No | 1 | 3 |  | Yes | 720 | | 1570 | | 77% | | 75% | | 38 | | 76% | |  | | 720 | | 1655 | | 81% | | 79% | | 40 | | 79% | |  |  |
| 02002 | Yes - From classes only | 1 | 2 | 12 | Yes | 180 | | 70 | | 3% | | 3% | | 4 | | 8% | |  | | 315 | | 610 | | 28% | | 29% | | 15 | | 31% | |  |  |
| 02004 | Yes | 1 | 2 | 13 | No | 270 | | 192 | | 9% | | 9% | | 8 | | 15% | |  | | 225 | | 150 | | 7% | | 7% | | 6 | | 13% | |  |  |
| 03008 | Yes - From classes only | 1 | 3 | 12 | Yes | 405 | | 147 | | 7% | | 7% | | 9 | | 18% | |  | | 405 | | 137 | | 7% | | 7% | | 9 | | 18% | |  |  |
| 01021 | No | 6 | 6 |  | Yes | 720 | | 1410 | | 84% | | 67% | | 36 | | 71% | |  | | 720 | | 1413 | | 84% | | 67% | | 36 | | 71% | |  |  |
| 01006 | Yes | 1 | 3 | 13 | No | 270 | | 0 | | 0 | | 0% | | 5 | | 9% | |  | | 270 | | 0 | | 0% | | 0% | | 5 | | 9% | |  |  |
| 01009 | No | 1 | 3 |  | Yes | 810 | | 1650 | | 81% | | 79% | | 41 | | 82% | |  | | 810 | | 1440 | | 71% | | 69% | | 38 | | 75% | |  |  |
| 01012 | No | 1 | 3 |  | Yes | 900 | | 395 | | 19% | | 19% | | 22 | | 43% | |  | | 900 | | 500 | | 25% | | 24% | | 23 | | 47% | |  |  |
| 01022 | No | 3 | 5 |  | Yes | 585 | | 1240 | | 69% | | 59% | | 30 | | 61% | |  | | 585 | | 1240 | | 69% | | 59% | | 30 | | 61% | |  |  |
| 01023 | No | 1 | 2 |  | No | 855 | | 1440 | | 67% | | 69% | | 38 | | 77% | |  | | 855 | | 2880 | | 133% | | 137% | | 62 | | 125% | |  |  |
| 01025 | No | 1 | 2 |  | Yes | 585 | | 1359 | | 63% | | 65% | | 32 | | 65% | |  | | 585 | | 1371 | | 63% | | 65% | | 33 | | 65% | |  |  |
| 01031 | Yes - From classes only | 1 | 2 | 13 | No | 540 | | 903 | | 42% | | 43% | | 24 | | 48% | |  | | 540 | | 908 | | 42% | | 43% | | 24 | | 48% | |  |  |
| 01034 | No | 1 | 4 |  | No | 180 | | 295 | | 15% | | 14% | | 8 | | 16% | |  | | 180 | | 295 | | 15% | | 14% | | 8 | | 16% | |  |  |
| 01035 | No | 1 | 2 |  | No | 900 | | 890 | | 41% | | 42% | | 30 | | 60% | |  | | 900 | | 1200 | | 56% | | 57% | | 35 | | 70% | |  |  |
| 01036 | No | 1 | 2 |  | Yes | 765 | | 1837 | | 85% | | 87% | | 43 | | 87% | |  | | 855 | | 2421 | | 112% | | 115% | | 55 | | 109% | |  |  |
| 01039 | No | 1 | 3 |  | Yes | 720 | | 1255 | | 62% | | 60% | | 33 | | 66% | |  | | 720 | | 1255 | | 62% | | 60% | | 33 | | 66% | |  |  |
| 01045 | No | 1 | 4 |  | No | 900 | | 2625 | | 137% | | 125% | | 59 | | 118% | |  | | 900 | | 2625 | | 137% | | 125% | | 59 | | 118% | |  |  |
| 01055 | No | 3 | 3 |  | Yes | 180 | | 180 | | 9% | | 9% | | 6 | | 12% | |  | | 180 | | 180 | | 9% | | 9% | | 6 | | 12% | |  |  |
| Totals | | | | | | | 12780 | | 24133 | |  | |  | | 615 | |  | |  | | 12960 | | 26845 | |  | |  | | 664 | |  | | |
| Total averages | | | | | | | 580.9 | | 1097 | | 54.4% | | 52.2% | | 28 | | 55.9% | |  | | 589.1 | | 1220.2 | | 60.1% | | 58,1% | | 30.2 | | 60.4% | | |
| Range | | | | | Max | 900 | | 3650 | | 179% | | 174% | | 75 | | 150% | |  | | 900 | | 3650 | | 179% | | 174% | | 75 | | 150% | |  |  |
|  |  |  |  |  | Min | 180 | | 0 | | 0% | | 0% | | 4 | | 8% | |  | | 180 | | 0 | | 0% | | 0% | | 5 | | 9% | |  |  |

^1^ Twenty-two dyads were observed during the classes and, of these, 15 dyads were interviewed (see corresponding column).
